# Endocarditis Caused by Bartonella quintana: A Case Report

**DOI:** 10.7759/cureus.78055

**Published:** 2025-01-27

**Authors:** Madalena Santos, Rita Figueiredo, Pedro Vasconcelos, Mariana B Nobre, Alba Acabado

**Affiliations:** 1 Internal Medicine, Centro Hospitalar Universitário Lisboa Norte, Hospital de Santa Maria, Lisbon, PRT; 2 Dermatology, Centro Hospitalar Universitário Lisboa Norte, Hospital de Santa Maria, Lisbon, PRT

**Keywords:** bartonella quintana, echocardiography, infective endocarditis, ischemic stroke, splenic infarct

## Abstract

Infective endocarditis (IE) is a complex and potentially life-threatening condition characterized by infection of the heart's endocardial surface, often leading to systemic complications. Historically recognized as a distinct pathological entity, IE has been associated with a wide range of bacterial pathogens, with *Bartonella* species emerging as a notable cause in recent years. Among these, *Bartonella quintana* is a rare but significant etiological agent, particularly in cases of culture-negative endocarditis. The management of *Bartonella*-induced IE remains challenging due to the evolving understanding of its pathophysiology and the need for tailored therapeutic strategies.

We present a unique case of a patient with *B. quintana* IE, remarkable for its simultaneous involvement of three cardiac valves - mitral, tricuspid, and aortic - each exhibiting vegetations. This multisite valvular involvement is an uncommon and distinctive feature, underscoring the aggressive nature of the infection. Notably, the patient's initial presentation was a massive ischemic stroke in the territory of the middle cerebral artery, an atypical manifestation of IE that highlights the potential for cardioembolic complications as the first clinical sign. Further evaluation revealed additional systemic embolization, including septic emboli to the spleen, further complicating the clinical picture.

This case underscores the importance of considering IE in patients presenting with embolic stroke, even in the absence of classic symptoms such as fever or heart failure. It also emphasizes the need for a high index of suspicion for *Bartonella *species in culture-negative endocarditis, particularly in cases with multisite valvular involvement and systemic embolic phenomena. Early diagnosis and targeted treatment are critical to improving outcomes in this rare but devastating condition.

## Introduction

*Bartonella* species are small, gram-negative bacilli responsible for a wide spectrum of diseases, ranging from cat scratch disease (CSD) and bacillary angiomatosis in immunocompromised patients to more severe conditions such as meningoencephalitis and infective endocarditis (IE) [1,2]. Among the 19 known species within the genus *Bartonella*, eight have been implicated in human IE: *Bartonella quintana, B. henselae, B. elizabethae, B. vinsonii *subsp. *berkhoffii, B. vinsonii *subsp*. arupensis, B. kohlerae, B. alsatica, *and* B. clarridgeiae* [3-5]. Of these, *B. henselae* and *B. quintana* are the most commonly associated with IE, accounting for the majority of cases [3,4]. These zoonotic pathogens are responsible for 1-15% of all IE cases and pose diagnostic challenges due to their fastidious nature, which often renders them undetectable by routine blood cultures [3-5].

*B. quintana*, first identified as the causative agent of trench fever during World War I, is transmitted primarily by the arthropod vector *Pediculus humanus corporis* (body louse) [6,7]. It is responsible for approximately 75% of *Bartonella*-related IE cases and is frequently associated with risk factors such as poor living conditions, inadequate hygiene, alcoholism, and human immunodeficiency virus (HIV) infection [7]. IE caused by *Bartonella *species can lead to systemic complications due to the embolization of fibrin-platelet vegetations from infected heart valves to distant organs, including the brain, lungs, kidneys, and spleen [8].

The diagnosis of *Bartonella* IE relies on a combination of clinical, imaging, and laboratory findings. Echocardiography remains the gold standard imaging modality for detecting valvular vegetations, with transesophageal echocardiography (TEE) demonstrating a sensitivity of 90-100% and specificity of 90-95% for diagnosing IE [3,9]. When combined with the modified Duke criteria, echocardiography can establish a definitive diagnosis [3,9]. However, given the challenges of culturing *Bartonella* species, serological testing plays a critical role in diagnosis. An IgG titer ≥1:800 against *B. henselae *or *B. quintana*, in conjunction with a compatible clinical history, strongly suggests *Bartonella *IE [10]. Advanced techniques such as western blotting with adsorbed sera can further differentiate between *B. henselae* and *B. quintana *infections [11]. Additionally, *Bartonella* species can be identified through tissue culture, agar plate inoculation, or visualization in valvular tissues using Warthin-Starry staining and immunohistochemical examination [12,13].

The treatment of *Bartonella *IE remains challenging due to the lack of standardized guidelines. Many patients have been successfully treated with empiric therapies for blood culture-negative endocarditis, which typically include a combination of β-lactam and aminoglycoside antibiotics. Surgical resection of the infected valve is often required in cases of severe valvular damage or refractory infection [1].

## Case presentation

A 65-year-old African man from Cabo Verde, who had been living in Portugal for 20 years, was brought to the Emergency Department (ED) following an acute onset of confusion and disorientation. The day prior to admission, the patient had visited the ED after experiencing a presyncopal episode accompanied by left hemiparesis, dysarthria, and labial commissure deviation. Although these symptoms gradually subsided, he left the hospital without formal discharge. The following day, he was found disoriented in the city center, exhibiting confused speech and amnesia for the event, prompting his return to the hospital.

Upon further inquiry, the patient was unable to provide detailed information regarding his medical history. However, his relatives reported a two-year history of progressive forgetfulness, though they denied any significant systemic symptoms such as fever, fatigue, or weight loss. The patient had a history of heavy alcohol consumption (approximately 17 units per week for over 30 years) and chewing tobacco use (six packs per year). He denied any other medical history, allergies, regular medications, or prior hospitalizations.

On physical examination, the patient was febrile with a tympanic temperature of 39°C and hypertensive (blood pressure: 157/93 mmHg). His respiratory rate was 18 cycles per minute, with an oxygen saturation of 99% on room air. Neurological examination revealed a conscious patient who was responsive to verbal stimuli but remained confused and disoriented in space and time. He exhibited difficulty performing simple tasks, a left-sided visual deficit, an unsteady gait with leftward deviation, decreased strength in the left arm (3/5), and left-sided spatial neglect.

Cardiac auscultation revealed rhythmic heart sounds with a systolic murmur most prominent in the aortic and mitral areas. Notably, the patient did not report or exhibit classic cardiac symptoms such as shortness of breath, orthopnea, or paroxysmal nocturnal dyspnea. Pulmonary auscultation demonstrated symmetrical vesicular breathing without rales or other abnormal sounds. Examination of the skin revealed scaly lesions on the lower limbs due to dry skin, as well as macular hyperpigmented lesions on the upper torso and palms. There were no signs of peripheral edema or other significant dermatological abnormalities.

Laboratory tests are described in Table 1.

**Table 1:** Laboratory parameters analysed in the ED ED: Emergency Department

Laboratory parameters	Value (units)	Reference value
Hemoglobin	10.2 g/dL	12.1-16.5
Hematocrit	44%	35-47
Mean corpuscular volume	85 fl	80-100
Mean corpuscular hemoglobin	30 pg	27-34
Leukocytes	10,000/mm^3^	3,500-10,000
Platelets	117,000/mm^3^	150,000-400,000
Creatinine	1.02 mg/dL	0.7-1.2
Blood urea nitrogen	42 mg/dL	17-43
Aspartate aminotransferase	31 U/L	8-33
Alanine aminotransferase	15 U/L	19-25
Gamma-glutamyl transferase	189 U/L	5-40
Total bilirubin	0.8 mg/dL	0.1-1.2
Lactate dehydrogenase	210 U/L	125-220
C-reactive protein	5.42 mg/dL	0.8-1
Immunoglobulin G	3,589 mg/dL	700-1600
Immunoglobulin A	468 mg/dL	70-400
Serum Immunofixation	Oligoclonal profile	

Serological tests for HIV, hepatitis B virus (HBV), hepatitis C virus (HCV), and syphilis were negative.

**Figure 1:**
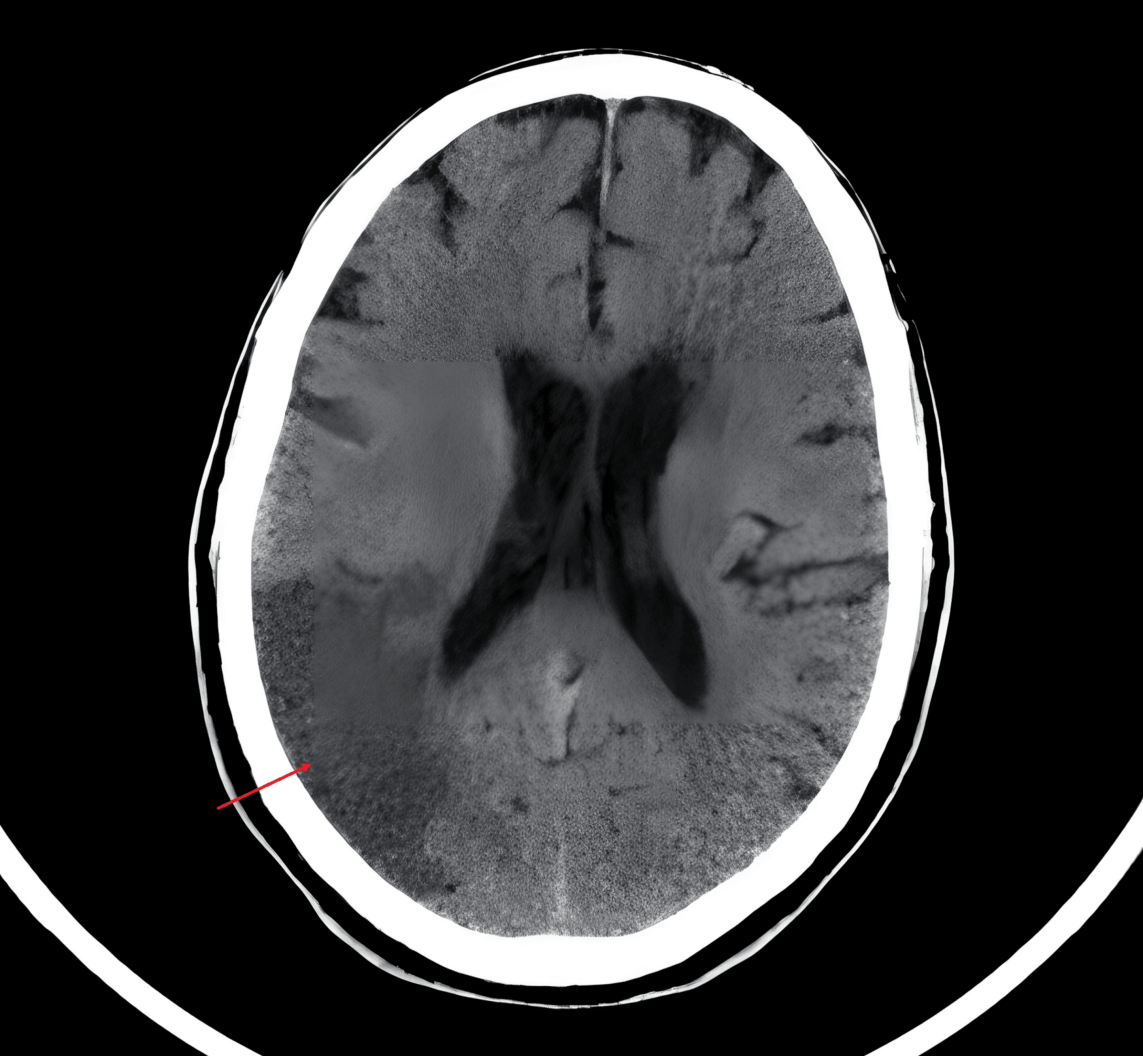
Axial image of the brain showing the right parietal cortical-subcortical hypodensity (red arrow), suggestive of an ischemic stroke as a cause of the patient's clinical findings.

A brain computed tomography (CT) scan revealed right parietal cortico-subcortical hypodensity extending from the high parietal convexity to the right posterior insular region and several hypodensities that were compatible with lacunar lenticular and cortico-subcortical sequelae (Figure 1).

The patient was admitted to the Department of Internal Medicine for further investigation of the findings described above. Given the acute neurological deficits and the absence of contraindications, antiplatelet and statin therapy was initiated for secondary stroke prevention. Empiric therapy with ceftriaxone and ampicillin was also started due to the initial suspicion of a central nervous system (CNS) infection.

Blood cultures were obtained upon admission, but no microorganisms were isolated. A lumbar puncture was performed, revealing clear cerebrospinal fluid (CSF) with normal cell counts, protein, and glucose levels. Microbiological assays of the CSF, including tests for cytomegalovirus (CMV), Epstein-Barr virus (EBV), *Treponema pallidum*, *Cryptococcus*, and *Borrelia*, were all negative.

**Figure 2:**
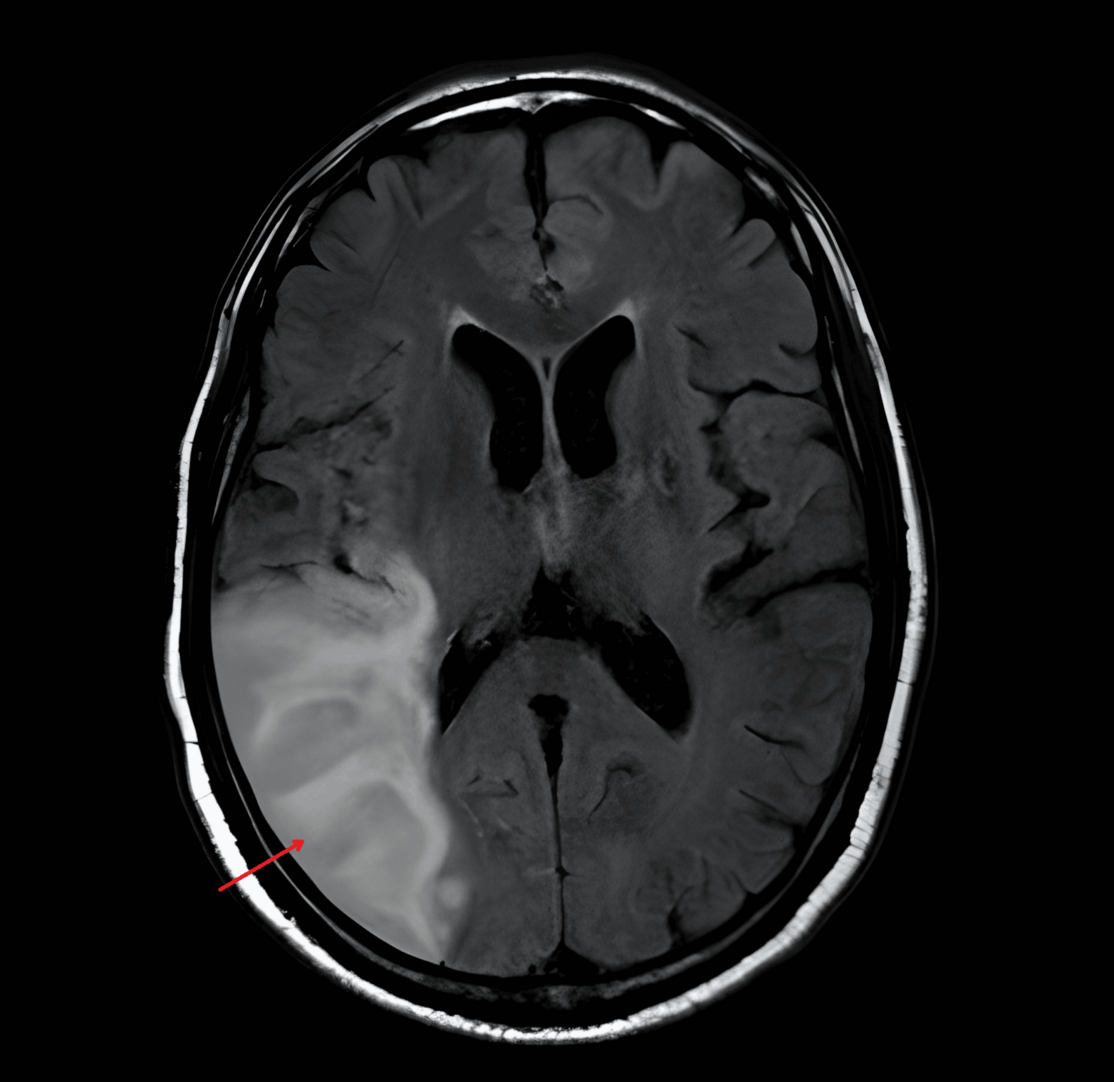
Axial image of the brain (T1-weighted) showing the right parietal cortical-subcortical hypodensity (red arrow). The magnetic resonance image confirmed the ischemic stroke in the territory of the middle cerebral artery.

**Figure 3:**
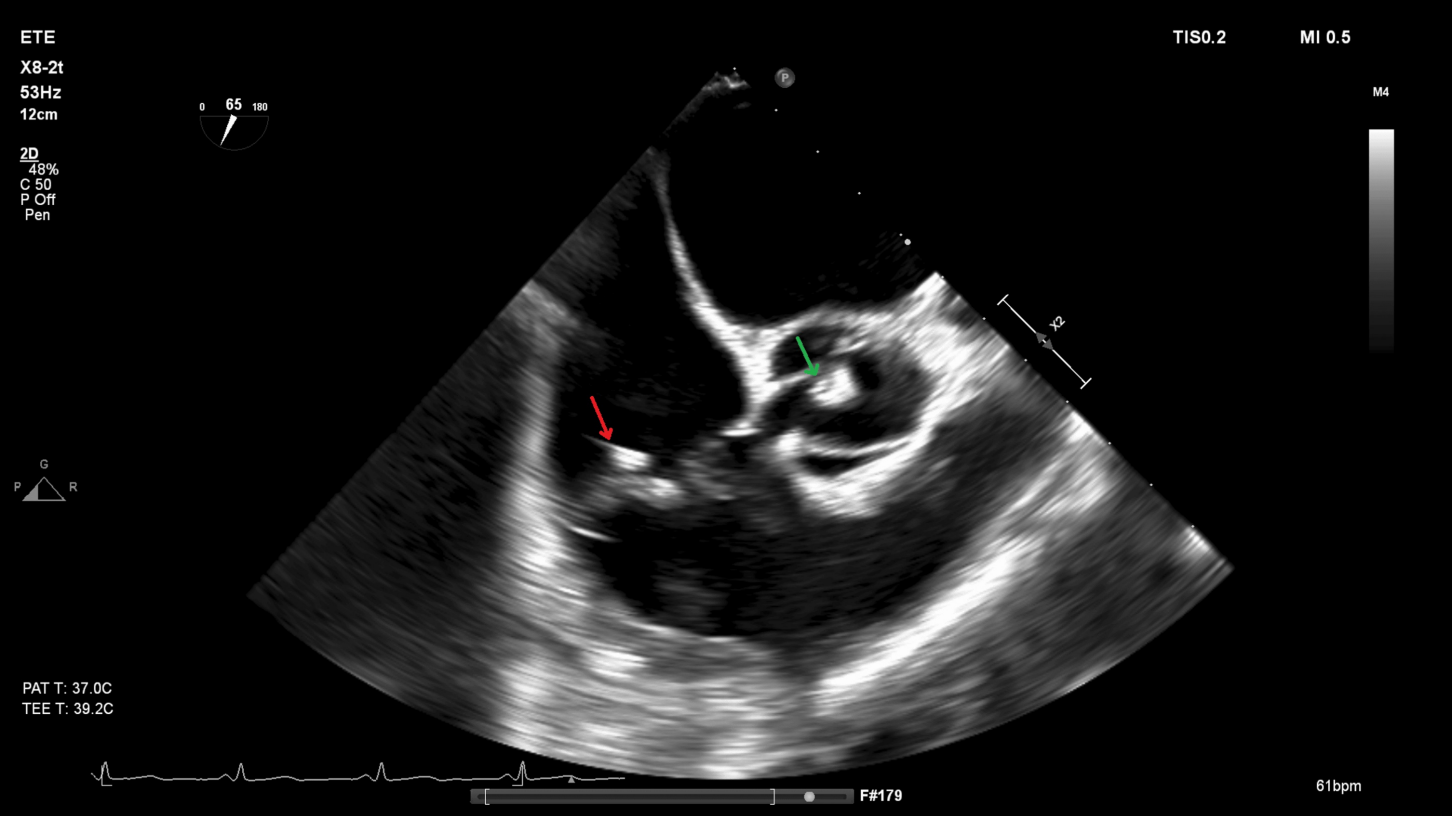
Transesophageal echocardiogram (short-axis view) showing vegetations on the tricuspid valve (red arrow) and the aortic valve (green arrow), which are the cause of the ischemic stroke.

During the hospital stay, additional diagnostic investigations were performed: a brain magnetic resonance imaging (MRI) that confirmed the presence of acute ischemic infarction in the territory of the middle cerebral artery, consistent with the patient’s clinical presentation (Figure 2); a Holter monitoring that excluded arrhythmias as a potential cause of embolic stroke; and a transthoracic echocardiogram (TTE) was performed on hospital day 3, due to the presence of a systolic murmur on physical examination and the clinical suspicion of a cardioembolic source for the stroke, which revealed vegetations on the aortic, mitral, and tricuspid valves, accompanied by valvular regurgitation. These findings were later confirmed by a transesophageal echocardiogram (TEE), which provided higher resolution and definitively supported the diagnosis of IE (Figure 3).

The TEE findings revealed moderate-to-severe regurgitation in both the mitral and tricuspid valves, with moderate eccentric regurgitation in the aortic valve. The presence of vegetations on all three valves - aortic, mitral, and tricuspid - suggested multifocal involvement. Notably, there was no clear evidence of valvular destruction or periannular extension, which might have indicated the local spread of infection. Instead, the simultaneous involvement of all three valves with distinct vegetations raised the possibility of independent seeding, a particularly unique and noteworthy presentation of IE. The aortic valve vegetation measured 11 mm and exhibited a complex morphology, with both nodular and filiform components. The mitral valve showed mild fibrocalcification and fibrosis but maintained good mobility, while the tricuspid valve had a 12 x 3 mm nodular vegetation on its anterior leaflet. These findings, combined with the absence of periannular complications, suggested that the infection may have originated from hematogenous spread rather than local extension.

The presence of a murmur on cardiac auscultation was a critical clue that prompted the echocardiographic evaluation. Despite the initial negative blood cultures, the multisite valvular involvement and the clinical context of a cardioembolic stroke raised strong suspicion for IE. This diagnosis was further supported by the absence of other identifiable causes for the patient’s symptoms.

At this point, the patient had one major criterion (echocardiographic findings) and two minor criteria (fever ≥38.0°C and vascular phenomena with an arterial embolism - ischemic stroke - and macular lesions in the palms of the hands, indicating Janeway lesions) that could attribute the cause of his condition to IE, according to the modified Duke criteria.

Hence, the antibiotic strategy was changed to gentamicin and ampicillin (with the completion of four days of ceftriaxone), assuming IE. All the repeated blood culture sets were negative.

**Figure 4:**
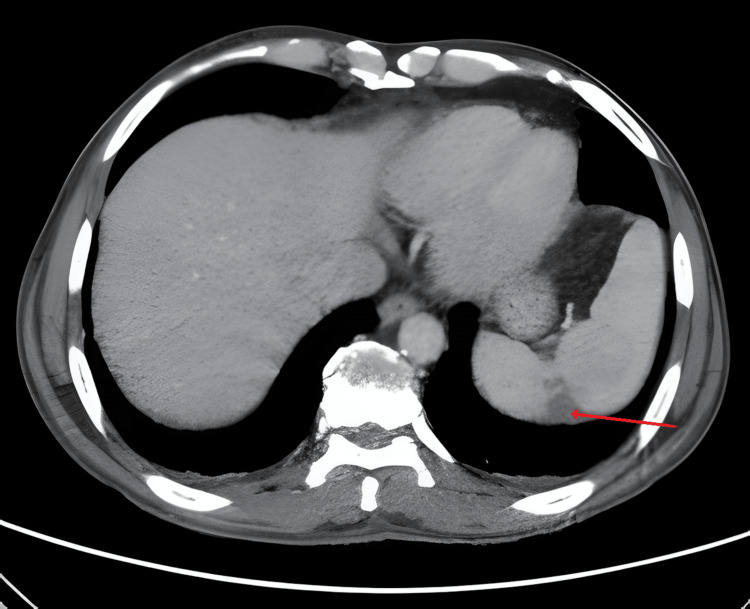
Axial image of the abdomen showing the splenic infarct (red arrow). The computed tomography image revealed a typical wedge-shaped area of low density in the spleen, suggestive of splenic infarct.

To rule out malignancy as a cause for marantic endocarditis, a thoracic, abdominal, and pelvic CT scan was performed, which excluded any signs of malignancy but revealed slight splenomegaly and the presence of a heterogeneous opacification of the splenic parenchyma, which represented a small area of peripheral infarction (Figure 4).

Additionally, an ophthalmology consult excluded any Roth spots at fundoscopy, and a skin biopsy was performed on one of the hyperpigmented lesions on the upper torso and limbs, which revealed post-inflammatory residual hyperpigmentation, with no signs of proliferative disease, and no microorganisms identified on the periodic acid-Schiff staining.

Since the blood cultures were negative, serologies for Q fever (*Coxiella burnetii*), brucellosis (*Brucella* spp., Huddleson reaction), and CSD (*Bartonella henselae*) were tested. The results showed an increased IgG titer for *B. henselae* of 1/4096, with negative IgM, and negative results for the others.

Considering the high titer for *B. henselae*, doxycycline was initiated and ampicillin was suspended.

**Figure 5:**
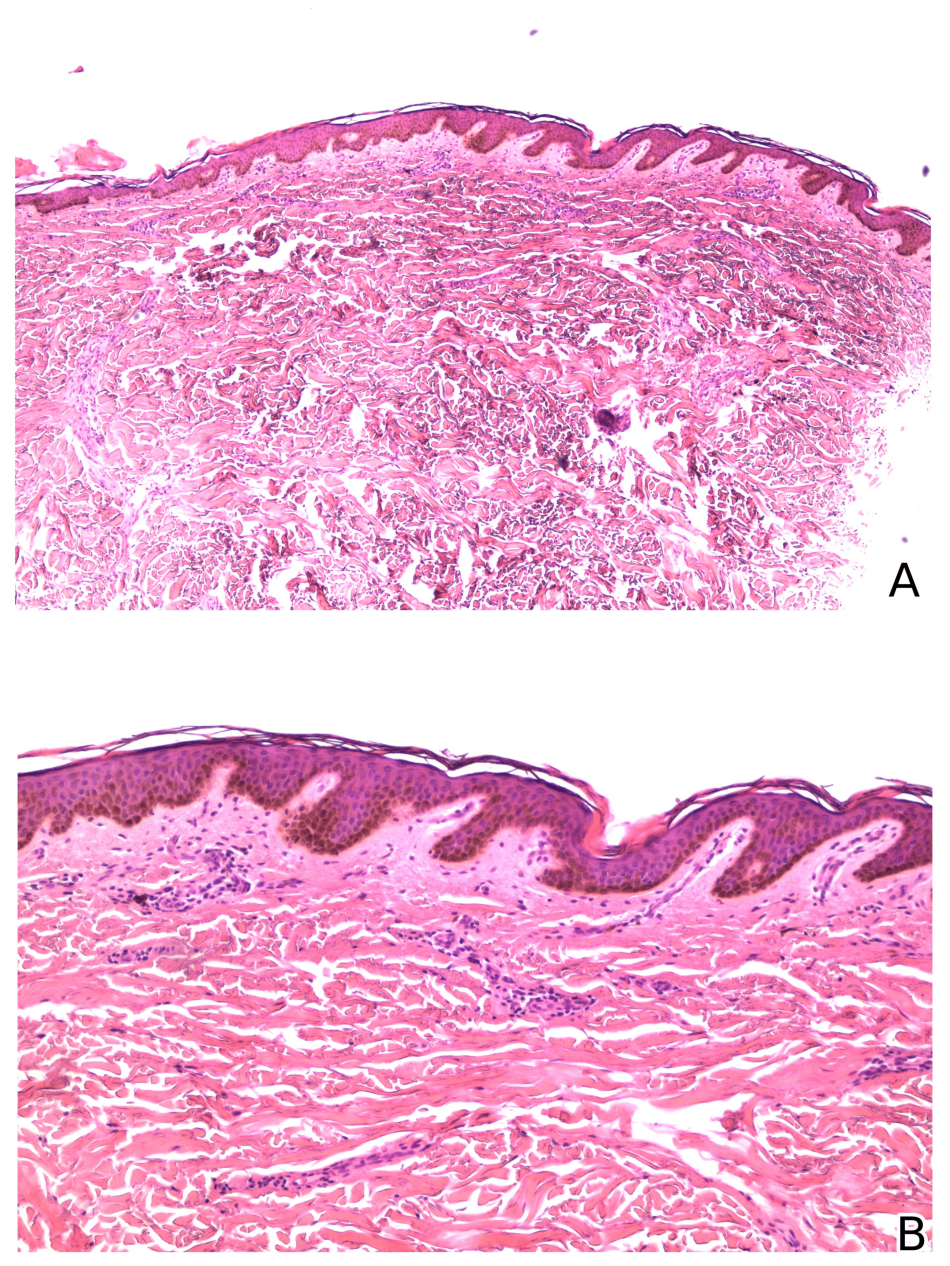
Histopathological analysis of the pigmented macular dorsal lesions. The histopathological analysis of the pigmented macular dorsal lesions revealed a normal epidermis, a slight inflammatory superficial perivascular lymphocytic infiltrate with some melanophages (A), and no signs of vasculitis, endoluminal septic thrombi, or proliferative disease (B).

In suspicion of bacillary angiomatosis, a skin biopsy was performed on the pigmented macular dorsal lesions, which revealed post-inflammatory residual hyperpigmentation, with no signs of active vasculitis or other vasculopathic changes, proliferative disease, and no microorganisms identified on the periodic acid-Schiff and Gram stainings (Figure 5). The lesions gradually regressed, not justifying a new biopsy.

The definitive diagnosis was confirmed by polymerase chain reaction (PCR) sequencing in the blood sample, identifying *B. quintana*, and so the patient continued doxycycline and gentamicin for 38 days with a positive response. PCR analysis for *Bartonella *in blood and CSF from the lumbar puncture at admission was negative.

Therefore, the patient was diagnosed with IE caused by *B. quintana *presenting as a cardioembolic stroke, and with septic emboli to the spleen, meeting one major criterion (echocardiographic evidence of endocardial involvement) and three minor criteria in the Duke's modified criteria (fever ≥38.0° C, vascular phenomena with an arterial embolism, Janeway lesions, and serologic evidence of active infection with an organism known to cause IE).

Unfortunately, during the patient's stay in the ward, he suffered a traumatic brain injury, which led to brain damage and subdural hemorrhage with intracranial hypertension and herniation, evolving into coma and culminating in his demise.

## Discussion

This case underscores the diagnostic and therapeutic challenges associated with IE caused by *B. quintana*. The patient’s initial presentation with acute neurological deficits was highly suggestive of an ischemic stroke, later confirmed by imaging. The concomitant presence of fever, altered mental status, and elevated inflammatory markers raised suspicion for a CNS infection, which was ultimately excluded by CSF analysis. The combination of ischemic stroke, systemic signs of infection (fever, elevated inflammatory markers), a systolic murmur in the aortic and mitral areas, and the presence of skin lesions on the palms guided the diagnostic team toward a possible diagnosis of infective endocarditis with systemic embolization to the brain, spleen, and small vessels of the skin.

An important differential diagnosis to consider in this case is marantic endocarditis (non-bacterial thrombotic endocarditis (NBTE)), which can present with similar clinical features, including valvular vegetations and systemic embolization. Marantic endocarditis is often associated with hypercoagulable states, such as malignancy or autoimmune diseases, and is characterized by sterile vegetations composed of fibrin and platelets. Unlike IE, marantic endocarditis typically lacks systemic signs of infection, such as fever or elevated inflammatory markers, and blood cultures are negative. In this case, the presence of fever, elevated inflammatory markers, and the eventual identification of *B. quintana* as the causative agent helped distinguish IE from marantic endocarditis. However, the overlap in clinical presentation highlights the importance of maintaining a broad differential diagnosis in patients with valvular vegetations and embolic phenomena [14].

The patient’s laboratory findings were consistent with the systemic inflammatory response seen in IE. The normocytic anemia was likely due to chronic inflammation, which impairs erythropoiesis and shortens red blood cell survival [15]. Hypergammaglobulinemia and an oligoclonal protein profile reflected immune system activation in response to the infection [16], while thrombocytopenia may have resulted from platelet consumption in vegetation formation and immune-mediated destruction [17].

The treatment of *Bartonella* endocarditis remains challenging due to the lack of standardized guidelines. While empiric therapy for blood culture-negative endocarditis often includes a combination of β-lactam and aminoglycoside antibiotics; the choice of doxycycline in this case was guided by the eventual identification of *B. quintana* as the causative agent. Doxycycline is a tetracycline antibiotic with proven efficacy against *Bartonella *species, particularly in cases of culture-negative endocarditis. It works by inhibiting protein synthesis and has excellent tissue penetration, making it a suitable choice for intracellular pathogens like *Bartonella*. In this case, gentamicin was initially added to provide synergistic bactericidal activity, as recommended for *Bartonella* IE, and was administered for 14 days. Doxycycline was planned for a minimum of six weeks to ensure eradication of the infection.

Given the extensive involvement of the aortic, mitral, and tricuspid valves, surgical intervention would have been necessary to repair or replace the damaged valves. However, the patient’s clinical deterioration precluded this option.

## Conclusions

This case highlights the diagnostic and therapeutic challenges of IE caused by *B. quintana*, emphasizing the importance of a comprehensive diagnostic approach in culture-negative IE. The uniqueness of this case lies in the simultaneous involvement of three cardiac valves - aortic, mitral, and tricuspid - each exhibiting vegetations, a rare and distinctive presentation that underscores the aggressive nature of *Bartonella* infections. Additionally, the patient’s initial presentation with a massive ischemic stroke, rather than the classic symptoms of fever or heart failure, further distinguishes this case and highlights the potential for atypical manifestations of IE. The evolving understanding of *Bartonella *species and their role in IE continues to refine diagnostic and therapeutic strategies, making it essential for clinicians to maintain a high index of suspicion, particularly in at-risk populations. Future research should focus on improving rapid diagnostic methods, optimizing treatment regimens, and developing prevention strategies for vulnerable populations to reduce the incidence and improve outcomes of this serious infection.
